# Compact characterization of liquid absorption and emission spectra using linear variable filters integrated with a CMOS imaging camera

**DOI:** 10.1038/srep29117

**Published:** 2016-07-08

**Authors:** Yuhang Wan, John A. Carlson, Benjamin A. Kesler, Wang Peng, Patrick Su, Saoud A. Al-Mulla, Sung Jun Lim, Andrew M. Smith, John M. Dallesasse, Brian T. Cunningham

**Affiliations:** 1 Department of Electrical and Computer Engineering, University of Illinois at Urbana-Champaign, Micro and Nanotechnology Laboratory, 208 North Wright Street, Urbana, IL, USA; 2 School of Electronic and Information Engineering Beihang University, 37 Xueyuan Road, Beijing, China; 3 School of Mechanical Science and Engineering Huazhong University of Science and Technology, Wuhan, China; 4 Department of Bioengineering University of Illinois at Urbana-Champaign Micro and Nanotechnology Laboratory, 208 North Wright Street, Urbana, IL, USA

## Abstract

A compact analysis platform for detecting liquid absorption and emission spectra using a set of optical linear variable filters atop a CMOS image sensor is presented. The working spectral range of the analysis platform can be extended without a reduction in spectral resolution by utilizing multiple linear variable filters with different wavelength ranges on the same CMOS sensor. With optical setup reconfiguration, its capability to measure both absorption and fluorescence emission is demonstrated. Quantitative detection of fluorescence emission down to 0.28 nM for quantum dot dispersions and 32 ng/mL for near-infrared dyes has been demonstrated on a single platform over a wide spectral range, as well as an absorption-based water quality test, showing the versatility of the system across liquid solutions for different emission and absorption bands. Comparison with a commercially available portable spectrometer and an optical spectrum analyzer shows our system has an improved signal-to-noise ratio and acceptable spectral resolution for discrimination of emission spectra, and characterization of colored liquid’s absorption characteristics generated by common biomolecular assays. This simple, compact, and versatile analysis platform demonstrates a path towards an integrated optical device that can be utilized for a wide variety of applications in point-of-use testing and point-of-care diagnostics.

The importance of providing point-of-care medical testing has been increasing rapidly as both the healthcare landscape and available technology have been in a period of significant change. Beyond human health diagnostics, point-of-use testing technology is needed for applications that include food safety, water quality monitoring and pathogen detection^1^,^2^. A large portion of the assays currently used for these applications are based on spectrally-resolved measurements of liquid-phase samples, where fluorescent emission and colorimetric absorption are the most commonly utilized techniques. For example, fluorescence spectroscopy is one of the most powerful tools for biochemical analysis, in which a fluorescent dye or quantum dot (QD) is used as a label to measure the presence and concentration of a specific analyte^3^,^4^. Likewise, absorption measurements are required for quantitative colorimetric tests that detect a change in color or optical density in a liquid sample, such as the widely used enzyme-linked immunosorbent assay (ELISA) for biomolecule analysis^5^. As point-of-care instrumentation emerges as a vital component for expanded access to biochemical diagnostics, the key barriers to entry for wide adoption of point-of-care devices remain portability, versatility, low cost, and ease of use^6^. The drive for a single, cost-effective optoelectronic platform that can perform a diverse array of biochemical analyses is indicative of the impact such a system would have on the field.

One approach for designing a miniaturized spectrometer is to replace the large diffraction grating and long monochromator path length with a small linearly-graded filter^7^,^8^,^9^. A linear variable filter (LVF) is made up of one or more dielectric cavities surrounded by distributed Bragg reflector (DBR) stacks of high-low index contrast, enabling highly selective and narrow bandwidth transmission. The thicknesses of the cavities are designed to incorporate a gradient along the length of the LVF to provide position-dependent wavelength selectivity for light incident perpendicular to the device. The transmitted wavelength is selected by the varying thickness of thin films in the dielectric stack, so that when the device is illuminated uniformly along its length, the intensity of light passing through the filter represents the spectrum of the incident light^10^. Early reports utilizing LVF spectroscopy have focused upon sensing the motion and fluorescence of an analyte^7^,^11^, measurement of the wavelength shift of resonant optical sensors^8^, and infrared absorptive detection with semi-compact platforms^12^,^13^. In a demonstration for gas sensing, two different desired IR detection ranges required are realized by implementing two LVFs specifically designed respectively onto a single IR detector^14^. Given that the spatial detection length of the system is limited by the size of the LVF, there is an inherent tradeoff between the spectral resolution and spectral range of the LVF^15^. With the length constraint, a wider spectral range implies lower spectral-spatial resolution while a higher resolution implies a lower spectral range.

By placing the LVF in front of a conventional image sensor array (such as a CCD, CMOS sensor array or position sensitive detector), as shown in Fig. 1, different wavelength components of the incident light can be spatially separated to illuminate different positions on the pixel array^7^,^8^,^11^. Compared to a spectrometer based upon a diffraction grating as the dispersive element, where a long propagation distance is necessary to achieve acceptable spectral resolution, a spectrometer based upon an LVF element has the advantage of compactness because it can be directly integrated onto a photodetector array, reducing the system’s size to that of a sensor chip. In spite of lower optical efficiency of an LVF-based spectrometer compared to a conventional spectrometer which obtains wavelength dispersion from a diffraction grating, the advantages of a compact and low cost system is of primary importance for instruments used for point-of-care testing. Utilizing a series of commercially-available LVFs instead of a single device allows for a broad spectral range detection system, while still maintaining high wavelength resolution.

In this paper, a simple and compact spectrum analysis platform, shown schematically in Fig. 1, is designed in which a set of LVFs is attached to a CMOS image sensor for fluorescence or absorption detection of a liquid-phase sample. The spectral range of the system is extended without sacrificing resolution by utilizing multiple LVFs simultaneously. While two LVFs are shown in this work, the approach can be extended to several LVFs arranged in parallel across the imaging sensor width, to cover a wide range of wavelengths, or specific wavelength bands with higher resolution. Performance of the system has been characterized and compared with a commercially-available portable spectrometer and a large-scale optical spectrum analyzer, demonstrating the capability and flexibility of this approach for general spectral analysis. Quantitative measurements are used to characterize the detection sensitivity of the system for several fluorescent analytes (semiconductor QDs and fluorescent dyes) and colorimetric absorbers. QD concentrations can be measured as low as 0.28 nM, while fluorescent dye concentrations as low as 32 ng/mL are observed. A commercially available water test for free chlorine concentration that generates a colored liquid as its output was performed, demonstrating the ability to detect chlorine concentrations as low as 3.9 mg/L. The quantitative experiments demonstrate acceptable performance for the applications in the area of point-of-care testing. Our experimental demonstrations show that, though spectral resolution of the LVF-based analysis system (Δλ = 3.77 nm FWHM at λ = 633 nm and Δλ = 6.09 nm FWHM at λ = 850 nm) is not as high as that obtained with the commercially available portable spectrometer (Δλ = 1.5 nm FWHM), it retains acceptable spectral resolving capability for sensing applications typically used in biological/chemical analysis with the added benefit of a high signal-to-noise ratio. With the characteristic of small signal detection ability, fine shape of the spectral profile, and the acceptable spectral resolution, this approach is suitable for analysis of emission and absorption spectra with broadband features, such as colorimetric dyes and fluorescent emitters used in most biological assays. This current approach maximizes the analytic abilities of a single CMOS image sensor by spanning both visible and IR spectra with multiple LVFs to enable quantitative measurement of the vast majority of biological assays within a single device. The approach demonstrated in this work provides a path towards a component design that can be inexpensively incorporated into mobile devices (e.g. smartphones) and further integrated with compact light sources (e.g. LEDs and semiconductor lasers) for general-purpose portable spectral analysis.

## Results and Discussion

### Characterization of the detection instrument

A helium-neon (HeNe) laser emitting a wavelength 632.8 nm was used to calibrate the visible (VIS) LVF with a spectral range of 400–700 nm, while a tunable infrared laser New Focus 6300 with tuning range from 835–855 nm was used to calibrate the near-infrared (NIR) LVF with a spectral range of 620–1050 nm. Using the spectra for each calibration source measured by the OSA (with a resolution of 0.05 nm), the linewidth of the HeNe laser is measured to be 0.11 nm and the linewidth of the tunable laser is 0.16 nm. Since the OSA resolution is not a limiting factor in determining the light source linewidths, these measured values accurately represent the actual linewidths of the sources under test and can be subsequently used to calibrate the spectral resolution of the LVFs. Using a HeNe laser illuminating the VIS LVF, the resolution (FWHM) is approximately 38 pixels at λ = 632.8 nm; similarly, the tunable laser on the NIR LVF has a resolution (FWHM) of 45 pixels at λ = 850 nm. The conversion from pixel number resolution to spectral resolution for the VIS LVF is calculated using three QD samples with visible emission spectra (QD520, QD560, QD610). Figure 2a depicts the emission spectra of each sample as measured by the portable spectrometer and Fig. 2b depicts the emission spectra as measured by the LVF system. The ratio of peak spacing in pixels for the three visible QD solutions from Fig. 2b to the corresponding peak spacing in wavelength from Fig. 2a is calculated to be approximately 10.07 pixel/nm for the VIS LVF. The spectral resolution of the visible LVF is approximately 3.77 nm, in good conformance with the expected 1–4% of wavelength. (1% = 6.33 nm) of the central wavelength from the VIS LVF specifications. The unit conversion ratio for the NIR LVF is calibrated utilizing a tunable laser, with wavelengths swept from 838 to 856 nm. A linear slope is calculated to be approximately 7.40 pixel/nm, which agrees well with the relation exhibited by the other two QD emission spectra (QD740, QD860). Utilizing this spatial-spectral conversion ratio, the resolution of the NIR LVF can be estimated as 6.09 nm, again conforming well to the expected 1–4% (1% = 8.5 nm) of the central wavelength from the LVF specifications.

Given the known tradeoff between spectral resolution and total wavelength range of an LVF with a fixed physical length, measurements were performed on a single CMOS image sensor with multiple LVFs, each operating over a different wavelength range. This enables an extended working spectral range of the LVF system while maintaining sufficient spectral resolution. Fluorescence measurements were performed on five separate QD samples with different emission wavelengths to demonstrate the wide operating wavelength range of the LVF system using the aforementioned measurement setup. Figure 2a plots the spectra obtained from the portable spectrometer, showing expected emission peaks for the QD solutions emitting near λ = 520 nm, 560 nm, 610 nm, 740 nm, and 860 nm. Figure 2b plots the emission spectra measured by the LVF analysis system demonstrating the capability of the device to perform spectral measurements over a wide range of wavelengths. For the QD samples with emission peaks at 520 nm, 560 nm and 610 nm, the region of analysis in the image captured from the CMOS sensor is limited to the portion covered by the VIS LVF; for the QD samples with emission peaks at 740 nm and 860 nm, the region of interest is similarly limited to just the portion covered by the NIR LVF. All the spectra obtained are plotted versus pixel number. Combining the measured data from the VIS and NIR LVFs allows for the extension of the functional range of the system while maintaining the spectral resolution of a single LVF. For spectral features such as QD emission or fluorophore emission that are wider than the resolution of the LVF spectrometer, spectra obtained are nearly identical to those obtained with a conventional spectrometer. The three emission peaks obtained with the VIS LVF appear to be broader in relation to the spectrometer than are the emission peaks obtained with the NIR LVF. This is in agreement with the fact that the VIS LVF has a larger spatial-spectral conversion ratio than the NIR LVF. Together, these results demonstrate the capability for the combined LVFs to detect fluorescent emission over a wide spectrum range with resolvable features, critical for fluorescent labeled biological assay detection.

### Quantitative emission measurements

A series of diluted fluorescent samples were tested in order to determine sensitivity and concentration detection limit. Diluted samples of the QD600 dispersion and infrared dye 800CW carboxylate solution were prepared for measurement using the VIS and NIR LVF portions of the platform as described in Materials and Methods.

For the VIS LVF measurement, six different concentrations of the QD600 dispersion were prepared ranging from 760 nM to 0.28 nM. The spectra measured with the VIS LVF are plotted in Fig. 3a, showing the stability in shape and emission detection across the range of concentrations. Analogous to increasing the sensitivity of a tabletop OSA through increasing the sweep time, the CMOS exposure time is increased as the concentration of the sample decreases to compensate for the reduction in total fluorescence emission. The measured signal intensities are then normalized by the exposure time to generate the final spectra, creating an approximate integration across spectral data for each concentration, the results of which are plotted along with the standard deviation whisker plot in Fig. 3b. A clear increase in total measured intensity with increasing concentration is observed, as expected.

For the NIR LVF, the spectra from concentrations starting at 20 μg/mL for the NIR dye are plotted in Fig. 3c. Since the excitation source is much stronger than the resulting fluorescence of the dye and much closer in wavelength to the maximum emission wavelength, a notch filter centered at λ = 785 nm was inserted between the cuvette and the sensor to block the source signal, explaining the dip in fluorescence emission intensity near the 800-pixel position. An integral approximation was performed for each concentration and plotted along with its standard deviation whisker plot in Fig. 3d. From Fig. 3c, it is shown that the LVF spectrum analysis system succeeds in detecting the fluorescence features of the NIR dye down to a concentration of 32 ng/mL.

We note that the CMOS camera has an adjustable maximum exposure time from 10 μs to 2000 ms for a fixed sensitivity and dark current. The exposure time was varied for different concentration samples to obtain a signal that does not saturate the sensor output. The exposure time used for the lowest concentration of QD600 dispersion, 0.28 nM was 2000 ms. A more sensitive imaging sensor would allow for measurements of even lower concentrations. For the NIR dye detection, the system is not limited by the CMOS sensitivity, but by the ability to suppress background illumination from the excitation source.

### Colorimetric absorption measurements

A series of absorption measurements were made for a variety of food dyes (red, orange, green and blue) in water. The wide and well-known absorption bands allow for the characterization of the LVF system over the entire operating wavelength range in just a few measurements. The light transmitted through the sample is detected using the LVF platform and compared to the spectrum of the white light source. Calculating the ratio of the sample transmission data for each color dye and the white light data results in the transmittance of the sample. Comparison measurements were made utilizing the portable spectrometer and the OSA, and all of the plots – transmission and transmittance – are shown in Fig. 4, where the curve color corresponds to the food dye color and the white light reference is plotted in black. In order to make a proper comparison with the raw data collected using the commercially available spectrometer and the OSA, the LVF data is presented as collected in Fig. 4c and the calculated transmittance is plotted in Fig. 4f, where both the measured pixel position and the corresponding wavelength are plotted in the same range.

As shown in Fig. 4, each setup presents benefits and drawbacks. Measurement with the portable spectrometer (Fig. 4a,d) is rapid, yet the detector response lacks sensitivity in the shorter wavelength region as compared to the LVF and the OSA. The reason lies in the relatively lower blue responsivity of the CCD sensor Sony ILX511B (*Sony, Tokyo, Japan*) in the Ocean Optics spectrometer (http://oceanoptics.com/wp-content/uploads/SONY-ILX511B.pdf). The OSA has the highest resolution due to its diffraction gratings and long optical path length, yet must be averaged over many measurements to reduce signal noise, thus requiring a larger and more expensive instrument with longer measurement time. Figure 4b demonstrates the small intensity signal noise inherent in the OSA measurement even after averaging 50 times. The spectrum obtained from the LVF system in Fig. 4c has been corrected by normalizing to the responsivity of the CMOS sensor in the system, and exhibits a smoother profile compared to the OSA spectra in Fig. 4b and has higher accuracy at shorter wavelengths than does the portable spectrometer spectra in Fig. 4a. It is noted that a few fringes are observable in the raw data collected with the LVF—in particular from the 1500–2700 pixel range—which are believed to be intrinsic to the LVF system. However, no additional calibration beyond the collection of the raw data and a reference measurement, which is taken before each set of samples (black curve, Fig. 4c), is needed to remove the fringes. As seen in the transmittance plot in Fig. 4f, these fringes disappear and the resulting curve represents a “fringe-free” interpretation of the data, which is smoother with better defined features compared to the curves generated from the measurements made using the portable spectrometer or the OSA. The comparison reveals that the LVF system is able to perform absorption measurements with a better signal-to-noise ratio while sacrificing minimal spectral resolution during absorption spectra measurement of colored liquids. Four different concentrations (five-fold dilution) of the green dye in purified water are used to demonstrate the capability of the system as a colorimeter, with the resulting transmission curves plotted in Fig. 5. Comparing the transmittance curves obtained from the two systems, it is noted that the LVF system loses little spectral resolution with less noise, confirming the increase in signal-to-noise ratio seen in the previous experiment.

Finally, the free chlorine test was performed, representing a frequently used quality measurement for municipal water supplies. The absorption spectra of the reference and the sample tap water, both treated with a DPD packet, were measured using both the portable spectrometer and the LVF system, as shown in Fig. 6. Defined as *A* = log_*10*_ *P*_*0 *_*/P*, where P_0_ is the original radiant power and P is the transmitted power after the absorption, the resultant data are presented as absorbance. By plotting the absorbance curve of the sample water over the original light through the reference, the results from the two systems are shown to agree well. Based upon the water quality report for the locality where the samples have been taken, Urbana, IL, the concentration of the free chlorine should be below 3.9 mg/L. Compared to these known values of chlorine present in city tap water, a good correlation between the known chlorine concentration and the chlorine absorption determined by measurements with the LVF-based system is seen.

## Conclusion

In conclusion, a compact and wide-range spectral analysis platform has been demonstrated using a set of optical linear variable filters directly mounted onto a CMOS image sensor array that can be utilized for liquid bioassay detection based on fluorescence emission and absorption measurements. The type of the CMOS sensor array is similar to arrays used in portable devices such as smartphones, creating the potential for integrating laboratory analysis capabilities into these devices. Multiple linear variable filters are integrated together onto a CMOS sensor array to extend the operating wavelength range while still retaining a high spectral resolution, and different regions on the CMOS sensor are selectable for different wavelength ranges of interest. Comparison with a commercially available portable spectrometer and a tabletop optical spectrum analyzer demonstrates that the analysis platform has a very good signal-to-noise ratio and acceptable spectral resolution. Dilution tests reveal that the fluorescence of a visible wavelength quantum dot can be detected at dispersion concentrations as low as 0.28 nM and fluorescence of a NIR dye solution was detectable at concentrations as low as 32 ng/mL. This same compact platform also shows free chlorine present down to 3.9 mg/L via absorption measurements. This LVF-based platform proves to be a useful and ideal method for point-of-care diagnostics, particularly bioassay detection based on fluorescence emission and/or absorption testing, for its compactness, reconfigurable nature, and the potential for further integration as a mobile optoelectronic device.

## Materials and Methods

### System components and wavelength calibration

Two LVFs (*JDS Uniphase Corporation, Milpitas, CA, USA*), one with a spectral range of 400–700 nm (VIS LVF) and the other with a spectral range of 620–1050 nm (NIR LVF) were attached side-by-side directly atop a CMOS camera (*PixeLINK, Ottawa, ON, Canada*) imaging chip. The sensitivity specification of the CMOS imaging sensor IBIS4-6600 (*ON Semiconductor, Phoenix, AZ, USA*) is 328 V*m^2^/(W*s), and the dark current is 3.37 mV/s. The exposure time of the CMOS camera can be adjusted from 10 μs to 2 s, as needed. The spectral resolution inherent in the LVFs was characterized by illuminating with single wavelength, narrow-linewidth sources, where a helium-neon (HeNe) laser emitting a wavelength 632.8 nm and a tunable infrared laser New Focus 6300 with tuning range from 835–855 nm (*Newport Corporation, Irvine, CA, USA*) were used respectively. The illumination spectrum of each calibration source was also measured by an optical spectrum analyzer (OSA) (*Yokogawa Electric Corporation (formerly Ando), AQ6315B, Musashino, Tokyo, Japan*) at its highest resolution of 0.05 nm to confirm the reference linewidth and wavelength. A Gaussian fit was applied to each spectrum obtained from the OSA, extracting the FWHM for the HeNe laser (0.11 nm at 632.8 nm) and the tunable laser (0.16 nm at 850 nm). An optical characterization system was constructed to collimate and expand the laser beam to fully illuminate the LVF-covered CMOS sensor. The camera software was adjusted to enable image capture, representing real-time measurement of the LVF transmissivity. A region of interest was selected from the captured image along the LVF gradient axis (Δ*x*), and averaged along the transverse direction to maximize the contribution from a row of 500 pixels representing a single wavelength. Because the illumination is provided at a single wavelength, while a band of pixels in the *x*-direction will register detected photon intensity, the achievable wavelength resolution of the LVF may be estimated by measuring the full-width at half maximum (FWHM) of the “peak” measured across the camera pixels. However, the pixel values from the camera must first be converted into their corresponding wavelength values.

The known emission spectra of the narrowband laser sources and liquid dispersions of fluorescent QDs were used to perform wavelength calibration of the system. For the VIS LVF, the pixel intensity distributions of three different QD samples with emission maxima ranging from 400 nm to 700 nm were measured using the LVF system and a portable spectrometer (*Ocean Optics, USB2000*+ *VIS-NIR, Dunedin, FL, USA*), producing a pixel-to-wavelength conversion table for the VIS LVF. A tunable infrared laser was utilized to calibrate the NIR LVF, where the operating wavelength was swept in 1 nm steps from 838 nm ≤ λ ≤ 856 nm and measured using both the LVF system and the OSA, producing the NIR pixel-to-wavelength conversion. The measured data from the LVF system and spectrometer/OSA were fit to Gaussian distributions and the extracted peak pixel position and wavelength values were plotted, resulting in a linear correlation between spatial and spectral units.

### Optical arrangements for emission and absorption tests

**Figure 1:**
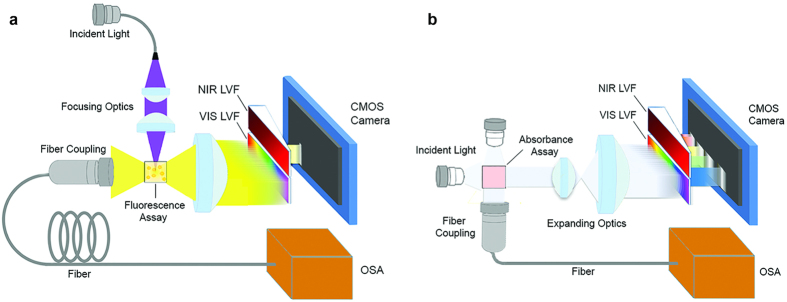
Schematic of the optical arrangements for the emission test (**a**) and the absorption test (**b**).

**Figure 2:**
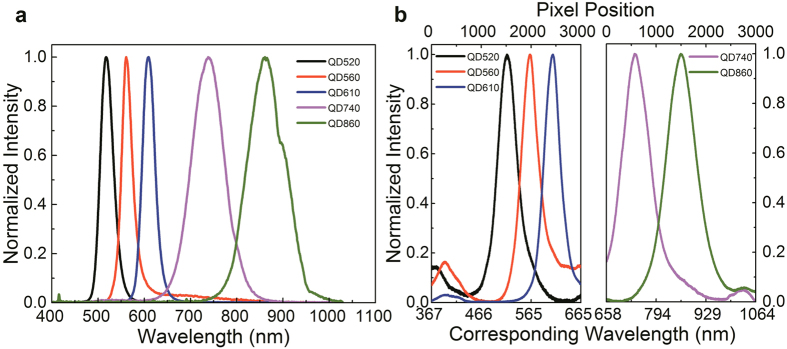
Fluorescence emission test for five different quantum dot dispersions. (**a**) Spectra measured with a portable spectrometer (**b**) Spectra measured with the compact LVF system, where QD520, 560, 610 are measured with the VIS LVF, and QD740, 860 are measured with the NIR LVF.

**Figure 3:**
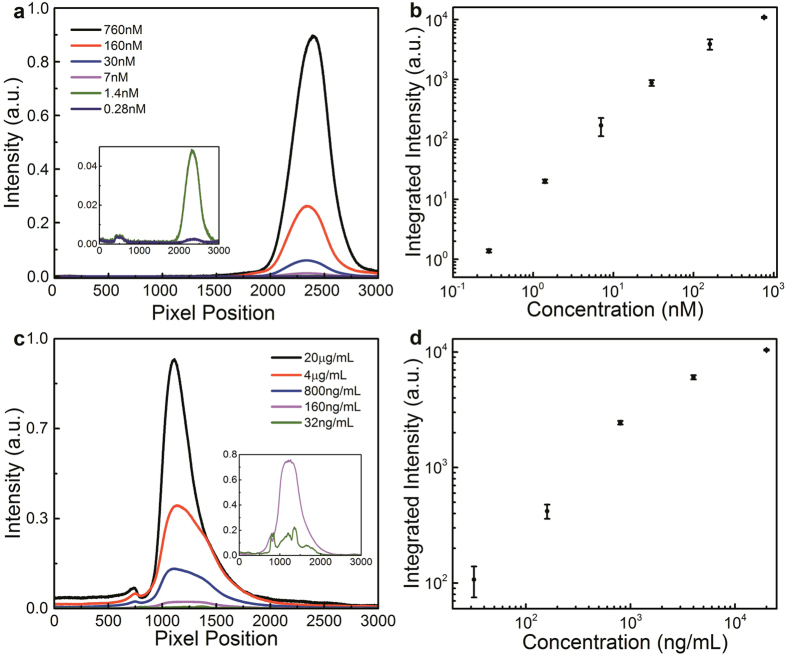
Quantitative fluorescence emission test with the LVF system. (**a**) Emission spectra of serial dilutions of QD600 samples using the VIS LVF. Inset shows the lowest two concentrations. (**b**) Integration of the emission spectra in (**a**) for each concentration, plotted along with the standard deviation whisker plot. (**c**) Emission spectra of IRdye solutions with different concentration for the NIR LVF. Inset shows the lowest two concentrations. (**d**) Integration of the emission spectra in (**c**) for each concentration, plotted along with the standard deviation whisker plot.

**Figure 4:**
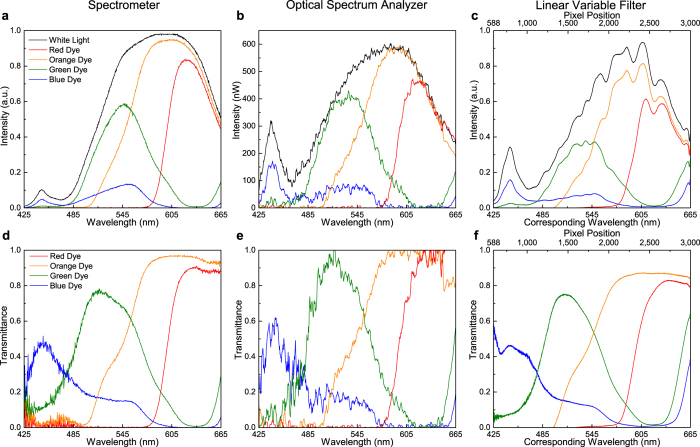
Absorption test comparison for different food dyes with three different systems. Transmission intensities of different food dye samples are measured with a portable spectrometer in (**a**), a tabletop OSA in (**b**), and the compact LVF system in (**c**). The curve color is corresponding to the color of the dye, and the reference of the white light incidence is plotted in black. The transmittance in correspondence is plotted in (**d**) for the spectrometer, (**e**) for the OSA and (**f**) for the LVF system respectively.

**Figure 5:**
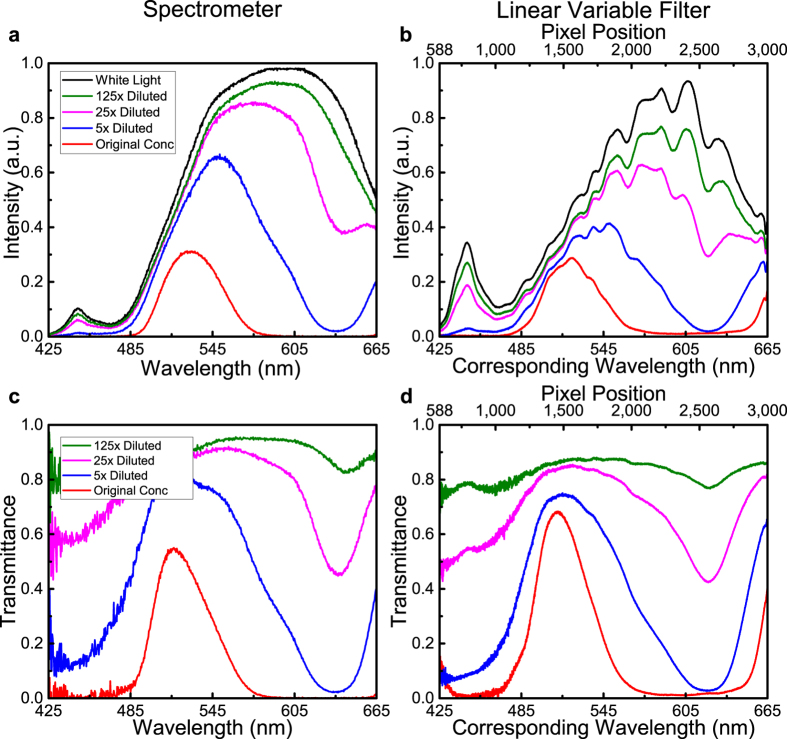
Absorption test for a serial of dilutions of the green dye. Transmission intensities of different concentrations are measured with a portable spectrometer in (**a**), and with the compact LVF system in (**b**). The transmittance accordingly is plotted in (**c**) for the spectrometer and (**d**) for the LVF system.

**Figure 6:**
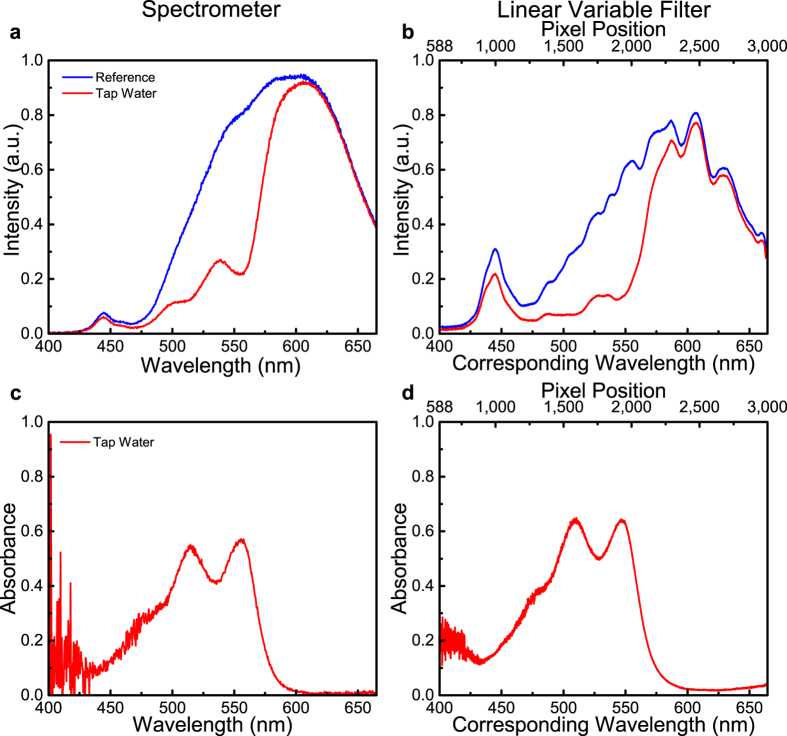
Absorption based water test for free chlorine. Transmission intensities of the reference (purified water treated with DPD packet) and the sample water (tap water treated with DPD packet) are measured with a portable spectrometer in (**a**), and with the compact LVF system in (**b**). The corresponding absorbance is plotted in (**c**) for the spectrometer and (**d**) for the LVF system.

Two separate optical component arrangements—one for colorimetric absorption measurements, the other for fluorescence emission measurements—were prepared, as shown in Fig. 1.

Fluorescence emission tests were performed across the full wavelength range of both LVFs. First, a series of QD dispersions with a range emission maxima (λ = 400–700 nm) were prepared for the visible LVF while two QD solutions with infrared emission (λ = 740 nm, 860 nm) and a solution of infrared dye 800CW carboxylate (*LI-COR Biosciences, Lincoln, NE, USA*) were prepared for the NIR LVF. All the QD samples were excited by a blue laser (*λ* = 410 nm) while the infrared dye was excited by a NIR laser that emits at *λ* = 785 nm (*Ocean Optics, Dunedin, FL, USA*). Each excitation source was focused into a 3.5 ml transparent quartz cuvette (*Starna Cells, Atascadero, 3-SOG-10-GL14-C, CA, USA*) containing the sample solution. The detection setup was arranged for the emission from the sample to be collected perpendicular to the excitation source path, and subsequently expanded and collimated to fully illuminate the entire surface area of the LVFs. For simultaneous measurement of the same liquid by a conventional instrument, an optical fiber placed on the opposite side of the cuvette from the LVF/CMOS sensor collected a portion of the same sample emission and directed it into the portable spectrometer.

The absorption tests were performed as shown in Fig. 1b. A set of white *miniTOPLEDs* (*OSRAM, Munich, Germany*) with a broadband emission spectrum extending from 400 nm ≤ λ ≤ 800 nm were combined in series to produce a beam that is collected and collimated via free-space optics into the solution under test within a transparent quartz cuvette. The transmitted light thus illuminated the sensing area of the LVFs. In order to obtain comparative data, the LVF attached CMOS camera was removed from the optical path. The transmitted light was captured by a 74-VIS fiber coupler *(Ocean Optics, Dunedin, FL, USA)* and then sent into the portable spectrometer through a QP400-2-VIS-NIR fiber (*Ocean Optics, Dunedin, FL, USA*) with a diameter of 400 μm and SMA connectors, or the OSA through a SMA to FC/PC fiber patch cable *(Thorlabs, Newton, NJ, USA)* with a diameter of 105 μm.

### Preparation of the test samples

Six different types of QDs were used for the fluorescence emission test. Five of the QD samples were synthesized in-house, and are named by peak emission wavelength designations: QD520, QD560, QD600, QD610, and QD740. The sixth QD type, QD860-OS, was purchased (*NanoOptical Materials, Inc., Carson, CA, USA)*. The QD synthesis was performed in high temperature organic solvents as described in detail in previous reports^16^,^17^. QD520, QD560, QD600, and QD610 were synthesized as core/shell/shell CdSe_y_S_1−y_/CdS/ZnS structures with emission color tuned by the core composition, and QD740 was synthesized as a core/shell Hg_x_Cd_1−x_Se/CdS structure. All nanocrystals were coated with aliphatic ligands, purified by acetone precipitation, and resuspended in chloroform. A small amount of oleylamine (~100 μL/3 mL) and tributylphosphine (~50 μL/3 mL) were added as additional ligands to enhance fluorescence intensity^18^. To study the concentration-dependent optical absorption spectra of QD600 core/shell QDs, a 0.73 μM QD600 stock solution (A_@350nm_ = 1.52) in chloroform was prepared along with a series of successive five-fold dilutions. Standard absorption spectra of the clear dispersions were measured using an Agilent Cary 5000 spectrophotometer (*Agilent Technologies, Santa Clara, CA, USA*), and the fluorescence spectra were obtained with a Horiba NanoLog fluorometer (*Horiba, Kyoto, Japan)*. Concentrations were measured using empirically determined extinction coefficients as described previously^17^.

Along with the QD samples, the near-infrared dye 800CW carboxylate used for fluorescence emission measurements was dissolved in PBS (phosphate buffered saline) solution, and a diluted series of samples were prepared in successive five-fold dilutions to generate a range of concentrations from 20 μg/ml to 32 ng/ml.

A set of household food colorings (*McCormick, Sparks, MD, USA*) were dissolved in purified water for initial characterization of colorimetric absorption sensitivity. To test limit of the detection in units of relative concentration, a single drop of green dye was added to 10 mL purified water to generate the highest dye concentration, and subsequently diluted five-fold and retested. The 5x dilution/measurement cycle was repeated until the LVF system could no longer register a change in transmission spectrum compared to the purified water calibration sample.

In order to test the capability of the system in the context of an analytical measurement, a commercially available assay kit was used to perform detection of free chlorine in water. DPD (N,N-diethyl-p-phenylenediamine) free chlorine reagent powder pillows (*Hach Company, Loveland, CO, USA*) were added to Milli-Q water (*Merck KGaA, Darmstadt, Germany*) to serve as a reference, while city tap water (Urbana, IL) was used for the test sample. Positive detection of free chlorine is indicated by the onset of a pink color, with intensity proportional to the chlorine concentration.

## Additional Information

**How to cite this article**: Wan, Y. *et al*. Compact characterization of liquid absorption and emission spectra using linear variable filters integrated with a CMOS imaging camera. *Sci. Rep.* **6**, 29117; doi: 10.1038/srep29117 (2016).
